# Response of a Blood Clot Adherent to Bone, Oral Mucosa and Hard Dental Tissues to a Uniaxial Tensile Test: An In Vitro Study

**DOI:** 10.3390/medicina60101673

**Published:** 2024-10-12

**Authors:** Gaia Pellegrini, Roberto Fedele, Daniela Carmagnola, Claudia Dellavia, Giorgio Pagni, Dolaji Henin, Gianguido Cossellu, Sabrine Fessi, Giulio Rasperini

**Affiliations:** 1 Department of Biomedical, Surgical and Dental Sciences, Università Degli Studi di Milano, 20100 Milano, Italy; gaia.pellegrini@unimi.it (G.P.); claudia.dellavia@unimi.it (C.D.); giorgio.pagni@gmail.com (G.P.); dolaji.henin@unimi.it (D.H.); giulio.rasperini@unimi.it (G.R.); 2 Department of Civil and Environmental Engineering (DICA), Politecnico di Milano, 20100 Milano, Italy; roberto.fedele@polimi.it; 3 Independent Researcher, 50100 Florence, Italy; 4 Independent Researcher, 20100 Milan, Italy; gianguido.cossellu@outlook.it; 5 Department of Periodontology, U.F.R. d’Odontologie-Garanciere, Paris and Rothschild Hospital (AP-HP), Université de Paris, 75006 Paris, France; sabrine_91f@hotmail.com

**Keywords:** blood clot, hard dental tissues, bone, periodontal tissues, wound healing, periodontal regeneration

## Abstract

*Background and Objectives*: Periodontal therapy aims to arrest the progression of periodontal diseases and possibly to regenerate the periodontal apparatus. To shift healing from repair to regeneration, the blood clot that fills the periodontal defect and remains in contact with structures such as tooth root, mucosa and bone needs to be stable, which is a reason why the treatment of non-containing periodontal bone defects, in which the clot may undergo displacement, is challenging. The gingival soft tissue, properly sutured, may act as a wall for blood clot stabilization. Knowledge on the response of the blood clot to stress and how it might vary according to the characteristics of the tissues it gets in contact with might be deepened. The aim of this study was to investigate in vitro, by means of a micro-loading device, the response of the complex formed by a blood clot and diverse tissues, simulating those involved in periodontal regeneration, to a displacing tensile test. *Materials and Methods*: Experimental samples made of two layers of either hard dental tissues, cancellous bone or oral mucosa, between which fresh blood was interposed, underwent a debonding experiment by means of a micro-loading device that measured their response to uniaxial tensile stress. *Results*: The peak of tensile stress and the overall work needed for the complete rupture of the clot’s fibrin filaments were significantly higher for hard dental tissues than for other tissues. However, mucosa sustained the highest maximal strain in terms of relative displacement between the plates of the micro-loading device to accomplish the complete rupture of the fibrin filaments compared to the other tissues, suggesting that the mucosa might act as a stable interface with the clot and be able to sustain tensile stresses. *Conclusions*: This in vitro study seems to support the use of mucosa to act as a wall for regenerative procedures of suprabony periodontal defects given its capability to form a stable interface with the clot.

## 1. Introduction

The periodontal apparatus is a very specialized anatomical entity composed of the alveolar bone, root cementum, periodontal ligament and gingiva. Periodontal diseases generally occur when bacteria trigger the periodontal complex, causing inflammation and a loss of support [1]. The principal goal of periodontal therapy is to arrest the progression of the condition by reducing the disease-causing bacterial load (cause-related therapy) and involving the patient in the re-establishment of adequate oral hygiene. Further treatment options, such as periodontal tissue regeneration (PTR), aim to promote the formation of a new periodontal attachment apparatus including its functions. If left untreated, periodontal diseases can lead to discomfort, tooth loss and related consequences, whose burden represents a remarkable individual and global issue [2,3]. For such reasons, deepening the knowledge on the biological mechanisms involved in periodontal disease progression and treatment options is important.

The loss of periodontal support can result in bone defects of various entities and morphologies adjacent to the involved tooth. During regenerative periodontal procedures, the blood clot that fills the surgically exposed bone defect needs to be populated by cells with the potential to produce a new periodontal attachment apparatus [4]. The residual bone and periodontal ligament have been proven to be able to provide stem cells with such characteristics [5]. Further, the blood clot owns intrinsic regenerative periodontal properties that can be displayed at their best when it is stable and adherent to the radicular tooth surface [6]. Therefore, the treatment’s success of bone defects largely depends, among other characteristics, on the morphology, entity and quality of the residual alveolar bone adjacent to the tooth, as they affect blood clot stabilization and stem cells supply [5]. Periodontal defects have been classified as “suprabony” when the base of the pocket is located coronal to the alveolar crest and as “infrabony” when the base of the pocket is located apically to the bone crest. Therefore, in suprabony defects, the vertical walls are mainly made of mucosa. For years, periodontal regeneration has been considered predictable only in the infabony component of a defect: the more self-containing the bone defect, the higher the regenerative potential [5]. PTR can be enforced by different means, including the interaction of bioactive molecules, cells and scaffolds [7]. Further, developing technologies and procedures, such as the employment of growth factors in the treatment of periodontal defects, is showing encouraging results, as they seem to positively affect wound healing towards full, multi-tissue periodontal regeneration [8]. Mini-invasive and customized surgical approaches of PTR are also under consistent development, with the goal to minimize the trauma connected to the access to the surgical area, reduce tissue inflammation and shift healing from reparative towards regenerative processes [6,8]. In this context, the stability of the clot itself in the regeneration of the attachment is gaining attention. The blood clot in a non-containing periodontal bone defect treated without space-maintaining barriers gets in contact with different structures, i.e., tooth root, mucosa and bone, and may undergo displacement since it is not protected by bony walls. While the importance of achieving blood clot stability within existing bone walls whenever possible is obvious, it has been suggested that the gingival soft tissue, properly sutured, even coronally to the bone crest, may act as a wall for blood clot stabilization—for example, in non-self-containing defects, replacing the use of space-maintaining devices or biomaterials [9]. To achieve such conditions, the flap and surgical approach should be designed by taking into consideration and trying to limit the stress operating on the clot, which can affect its adherence to the mentioned tissues and impair the treatment outcome, that is, stabilizing as much as possible the blood clot within the periodontal defect through the management of precise flap design and sutures.

The mechanical behavior of the blood clot has been studied in several fields, mainly in cardiovascular medicine, concerning different aspects such as its dissipative mechanics under uniaxial extension, cyclic loading and stress-relaxation, the effect of shear stress on platelets, the mechanical characterization of clot analogs under both tensile and compressive loading and stress-induced hypercoagulation [10,11,12,13].

In the periodontal setting, qualifying and quantifying the response of the blood clot to stress and how it might vary according to the characteristics of the tissues it gets in contact with might help improve the outcomes of periodontal regeneration in terms of the predictability of the procedures and strategies concerning the use of biomaterials and/or devices [8]. Further, it may help define flap design and suture techniques capable of protecting the clot in the best possible way to stand stress in different clinical situations, as it is known that occlusal forces on the tooth are spread to the healing area and negatively influence regenerative procedures [14].

For these reasons, the present study aimed to investigate in vitro how different experimental samples characterized by a blood clot interposed between different tissues (bone, mucosa and hard dental tissue), mimicking those involved in its stabilization during periodontal healing, responded to uniaxial tensile stress (TS).

The null hypothesis of the study was that there would be no difference in the tensile stress peak generated during the debonding experiment between mucosa (simulating the wall of a suprabony defect) and bone (simulating the wall of an infrabony defect).

## 2. Materials and Methods

### 2.1. Study Design

The study was approved by the Institutional Review Board (or Ethics Committee) of Università degli Studi di Milano (protocol number 107/20 of 17 November 2020).

**Figure 1:**
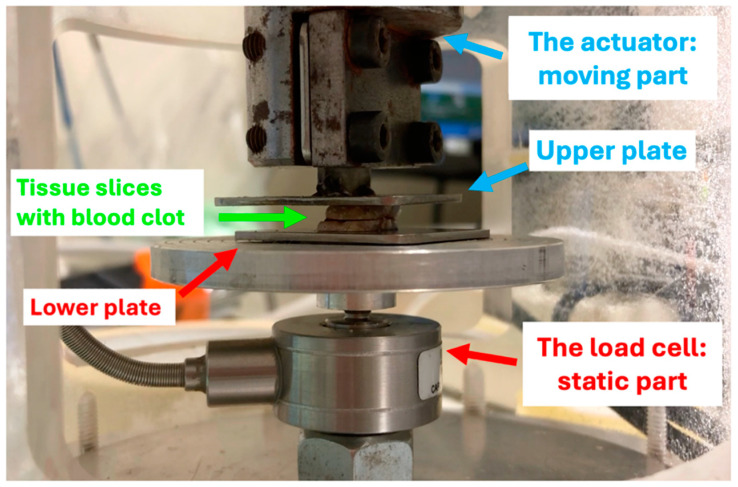
The micro-loading device.

A micro-loading device for the mechanical debonding (debonding experiment, DE) of previously assembled blood clot–tissue complexes (BCC) was developed (Figure 1).

The micro-loading device consisted of a thick cylindrical chamber of poly(methyl methacrylate) (PMMA) containing a piezoelectric linear actuator (moving part) and a load cell (static part) (Figure 1). The piezoelectric linear actuator (N-216 NEXLINE^®^ PiezoWalk^®^ by Physik Instrument, Bresso, Milan, Italy), fastened to the upper part of the apparatus, was apt to induce a uniaxial relative displacement (normal separation with respect to the clot) with a maximum stroke of 20 mm, a resolution of about ±4 nm and withstand loading up to 60 kg. The vertical displacement of the actuator was remotely controlled via a digital controller and commercial software (SM148E Software Manual PIMikroMove™ Release: 2.4.0, 2009, Physik Instrumente (PI) GmbH & Co. KG, Karlsruhe, Germany).

The miniature tension and compression load cell (WMC series, maximum capacity 12 kg, accuracy 0.15% Full-Scale Output produced by Interface Inc., Scottdale, PA, USA) was located in the lower part of the chamber. A central screw fastened it to the rigid basis of the apparatus. An acquisition card (National Instruments NI-DAQ USB 6251, Series 16-Bit, 1.25 MS/s, Austin, TX, USA) allowed the user to acquire the electric signal provided by the load cell remotely on a computer within a LabView environment, where experimental data were monitored and stored.

The slice holders, namely, the upper and lower plates, were placed in the center of the PMMA chamber, between the actuator and the load cell, and the grips were designed for the specific sample geometry.

During the experiment, the actuator’s displacement generated a monotonically increasing relative displacement orthogonally directed to the reciprocally facing slices’ surfaces and the interposed clot, which behaved as an adhesive joint subjected to peeling (mode I). The load was detected by the micro-load cell, which, in response, provided the electric signal that was acquired for the investigation.

To mimic the tissues involved in the formation of the walls of periodontal defects, three types of BCC were developed, each consisting of either two layers of hard dental tissues, cancellous bone or oral mucosa, between which fresh venous blood was interposed.

The tissues were obtained as follows:

- Hard dental tissues: 8 patients from a private clinic, scheduled for tooth extraction for periodontal reasons, provided 11 intact teeth (5 canines and 6 premolars) from which hard dental tissues were obtained. The teeth had to be free from fillings or decays. Each patient signed an informed consent form.
- Cancellous bone: 22 samples of cancellous bone were obtained from fresh bovine ribs provided by a local butcher the day of the experiment.
- Oral mucosa: Oral mucosa was harvested from the inner side of the same animals’ cheeks.
- Blood: Fresh blood was obtained from one healthy volunteer (GP) by means of a capillary finger prick.

The tissue samples were processed as follows:

- After extraction, the teeth were placed in saline solution and used within 24 h. For each tooth, two parallel slices that were 1.5 mm thick and of similar areas were cut by means of a bone microtome and ground to their final thickness (Remet, Bologna, Italy) (hard dental tissues group);
- Parallel slices (about 3 mm thick and 1 × 2 cm^2^ wide) of cancellous bone were cut from the bovine ribs by means of a bone microtome (Remet, Bologna, Italy) and used within 24 h from delivery (bone group);
- Oral mucosa slices from bovines (about 3 mm thick and 1 × 2 cm^2^ wide) were cut by means of a surgical blade and used within 24 h from delivery (mucosa group);
- Capillary human blood was collected by a micro-pipette at the time of the experiment and used immediately.

The cancellous bone and oral mucosa slices were cut at dimensions matching those of the sliced teeth. Each slice was photographed frontally by a calibrated digital camera (D3500, Nikon, Tokyo, Japan), and its area was assessed by means of an image analysis system (Image J, NIH, Bethesda, MD, USA).

#### Sample Size Calculation

Sample size calculation was performed considering the variable “displacement at final blood clot fracture” in the following equation [15,16]:

{2(*Z_α_*_/2_ + *Z_β_*)^2^*σ*^2^}/*δ^2^*

using *α* = 0.05 and the power of sample (1 − *β*) = 80%. For the variability (*σ* = SD), the value of 5.19, obtained in a previous paper, was considered [16]. The minimum significant value (*δ*) in terms of the periodontal defect depth to be regenerated was set at 8 mm. On the basis of such data, the number of samples to be used in the present study was 11 for each group.

### 2.2. Debonding Experiment

The DE was carried out in a room with a constant temperature of 21 °C and a relative humidity of 50% and always from the same operator (RF) to guarantee repeatability.

**Figure 2:**
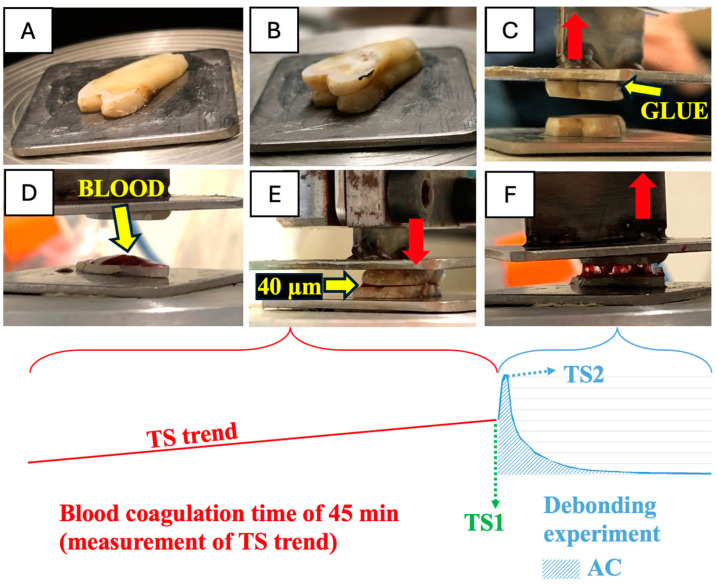
(**A**) The first slice was glued by a drop of acrylic resin to the lower flat plate connected to the load cell of the machine; (**B**) The second slice was positioned on the top of the first one and vertically aligned; (**C**) The second slice was glued to the upper plate of the machine fixed to the actuator and was then moved up to create space between the slices; (**D**) The upper surface of the lower (first) slice was completely covered by 1 microliter of blood; (**E**) The upper (second) slice was moved down to reach 40 μm from the lower slice and left in this position for 45 min (BCT). (**F**) After coagulation, the vertical displacement of the actuator was started. TS: tensile stress measured at the end of the coagulation time (TS1) and at its peak during the debonding experiment (TS2); AC: area under the curve. The red arrow indicates the direction of the movement.

Each DE was carried out using two tissue slices of the same tissue. In the micro-loading device, the lower plate was fastened to the load cell (static part), and the upper plate was fixed to the actuator (vertically moving part). Then, the first slice was glued to the lower plate of the machine with a drop of acrylic resin by one operator and left in place for one minute, until the glue was set. The second slice was carefully positioned on top of the first one, ensuring vertical alignment (Figure 2A,B).

After the two slices were aligned, the upper surface of the second slice was covered with a drop of the same acrylic glue, the down-moving actuator moved to reach it and it was left to rest for one minute. This position of the upper plate was considered as the reference point for calibrating further movements. At this point, the upper plate was moved up (Figure 2C). When the two slices were sufficiently far from each other, the upper surface of the first slice was completely covered by one microliter of blood (Figure 2D).

At this point, the upper (second) slice was moved down to reach 40 μm from the lower slice (considering the reference point): this distance was intended to allow the blood clot to completely fill the space between the slices and cover their two free faces (Figure 2E). This moment was recorded as T0, and from this time, the ES was left to rest for 45 min [17] (blood coagulation time, BCT).

The load cell signal was monitored throughout the entire BCT, ensuring a comprehensive understanding of the experiment’s progress. After this time, the vertical displacement of the actuator was started, with a constant speed of 30 mm/s, up to the complete debonding of the slices (Figure 2F).

Video S1 available in the Supplemental Materials Section illustrates the described process.

### 2.3. Measurements and Statistics

#### 2.3.1. Measurements

The outcomes of the present study refer to the response of the complex formed by the blood clot and hard dental tissues, bone and oral mucosa to a displacing TS. This response was constantly recorded during the entire BCT and during the DE, until the blood clot ruptured and the reciprocally facing slices were wholly separated. For each DE (11 per each tissue type), TS recording and analysis were used to achieve normalized parameters by dividing the row TS data by the mean area of the two reciprocally facing slices’ surfaces, as follows:

- rAC, the normalized area under the curve (AC) formed by the TS generated during the DE (Figure 2). This value was computed to assess the overall TS generated in the debonding activity and represents the separation work generated by the BCC during the debonding process;
- rTS1, the normalized TS detected at the 45th min of the coagulation time, immediately before the vertical displacement (TS1): the local and spontaneous TS generated by the blood clot at the end of the BCT as an indicator of the mechanical effects of coagulation in terms of progressive shrinkage;
- rTS2, the normalized TS detected during the DE at the peak force as an indicator of the local strength of the BCC, generated during the debonding process (TS2);
- RP, the distance (w) travelled by the upper plate up to the blood clot rupture induced by the stroke-induced displacement (normal displacement or separation at the joint).

#### 2.3.2. Statistical Analysis

Raw data were collected and stored in a Microsoft^®^ Excel file (version 16.89). Descriptive statistical analyses were performed using the Microsoft^®^ Excel program, while inferential statistics were performed using the IBM^®^ SPSS^®^ software platform (version 27).

Descriptive statistics (mean, standard deviation—SD) were expressed for each parameter (rAC, rTS1, rTS2 and RP) separately for every tissue type.

To assess the normal distribution of all measurements within each tissue group, skewness and kurtosis were calculated. No large deviations from normality were found: the skewness ranged between +1.51 and −0.63, and kurtosis ranged between +2.93 and −1.58.

The differences between groups were computed by an unpaired Mann–Whitney test. A *p* < 0.05 was considered significant.

## 3. Results

Eleven pairs of reciprocally facing slices were prepared for each tissue typology. The average value of the surface area was 122.4 ± 36.1 mm^2^ for hard dental tissues, 222.1 ± 73.58 mm^2^ for trabecular bone samples and 341 ± 17.5 mm^2^ for mucosa.

### 3.1. Debonding Experiments

Eleven DE for each tissue (one test for each pair of slices of the same tissue, namely, on hard dental tissues, bone and mucosa slices, all joined by a blood clot) were performed separately.

**Figure 3:**
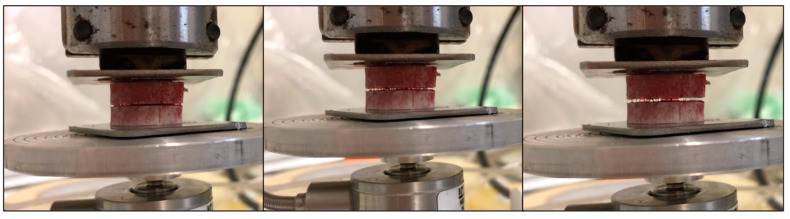
Blood clot filaments have formed between two bone samples. The fibrin filaments become thinner while the distance between the facing surfaces increases, until rupture occurs.

At the observation, at the end of the BCT, the clot appeared firmly located between the slices without extravasation. During the DE and the progressive displacement of the upper plate, the blood clot remained adherent to both surfaces, and fibrin filaments appeared to elongate between the facing surfaces. While the space between the plates increased, they became progressively thinner and fewer in number until rupture occurred (Figure 3).

### 3.2. Measurements

Descriptive analysis: During the BCT (45 min), for all experimental samples, the load cell detected a spontaneous and progressive increase in the mutual TS acting between the two slices bonded by the clot. This force was maximal at the end of the BCT (TS1). Table 1 reports the values derived from the normalization and analysis of the TS data detected at the end of the coagulation time (rTS1) and at the peak of the DE (rTS2). The table also reports the distance travelled by the upper plate up to the blood clot rupture (RP).

#### Inferential Analysis

The analysis with a Mann–Withney test provided that the rTS1 and rTS2 data of hard dental tissue were significantly higher than those of both bone and mucosa (*p* < 0.05), and those of bone were significantly higher than those of mucosa (*p* < 0.005).

The rAC values of hard dental tissues were significantly higher than those of both bone and mucosa (*p* < 0.05), while no differences emerged between the rAC values of bone and mucosa.

The RP values of mucosa were significantly higher than those of both bone (*p* < 0.005) and hard dental tissues (*p* < 0.05), and bone displayed significantly higher values than hard dental tissues (*p* < 0.001).

### 3.3. TS Curves during the DE

**Figure 4:**
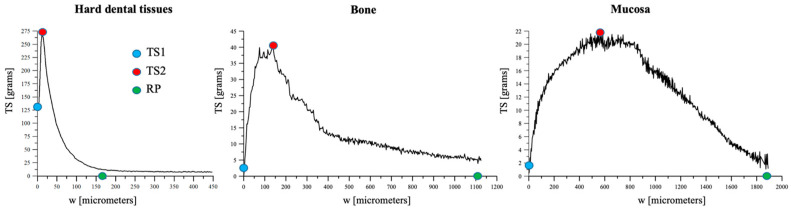
Mechanical response of the blood clot–tissue complexes in terms of relative TS versus normal separation (i.e., vertical relative displacement (w) for hard dental tissues (I), bone (II) and mucosa (III)). TS is represented at the 45th minute, immediately before the vertical displacement (TS1), at the peak force (TS2) and at RP.

The debonding curves help visualize the mechanical response of the BCC for hard dental tissues (I), bone (II) and mucosa (III) in terms of TS versus normal separation (i.e., vertical relative displacement (w) (Figure 4).

Marked differences appeared in the morphology of the curves among the different BCCs (Figure 4). Hard dental tissues exhibited an approximately linear response up to the peak, followed by a rapid nonlinear decrease in its bearing capacity and an early rupture point. The peak stress for hard dental tissue was significantly higher with respect to bone and mucosa. The response of mucosa was characterized by a marked nonlinear response since the beginning (TS1) was close to the origin (value 0). Its curve appeared jagged and round, with a peak stress (TS2) delayed and about ten times lower than that of hard dental tissue and half of that of bone. Bone exhibited intermediate behavior, in terms of TS1 and TS2, RP and work of separation, displaying a progressive trend both in reaching the peak as well as in approaching the RP.

## 4. Discussion

The findings of the present study show that the three tissue typologies we tested provided different responses in terms of the TS trend during the DEs. The peak of TS (rTS1) and the overall work needed for the complete rupture of the fibrin filaments of the blood clot (rAC) were significantly higher for hard dental tissues than for the other tissues. However, mucosa showed the highest maximal travelled distance (RP) of the upper plate to accomplish the complete rupture of the fibrin filaments compared to the other tissue typologies. When translated into a clinical context, these data may be suggestive of the maximal dislocation that a blood clot may bear before undergoing modifications that may affect its physiological role in regenerative processes. Such results may support the use of soft tissues as a suitable wall within the periodontal defect during regenerative procedures, as mucosa can compensate for movements of the flap. On the other hand, the sudden debonding after peak stress and the lower RP for hard dental tissues (277 µm) than for mucosa (1351 µm) and bone (610 µm) may underline the need to stabilize the tooth before regenerative surgery to prevent fibrin filaments debonding from the tooth root, which may in turn lead to the creeping growth of the junctional epithelium (reparative healing) rather than to fibrin filament remodeling into new periodontal attachment (regenerative healing) [18].

According to the results of the present study, mucosa seems to bear and accommodate large displacements without breaking, whilst hard dental tissue samples exhibit a brittle response, as, after the peak stress, sudden failure occurs with a rapid decrease in their loading capacity. The maximum vertical displacement travelled by the upper sample until the complete rupture of the fibrin filaments (RP) derives from the superposition of the elastic deformation of the tissue slices and the hardened clot that filled the space joining the internal surfaces of the samples. As a reference, reliable values of Young’s elastic modulus derived from the literature are 30 GPa for peritubular and about 19 GPa for intertubular dentin (I), 14 GPa for cortical bone, 0.5 GPa for cancellous bone (II) and about 8.0 × 10^−5^ GPa for mucosa (III) [19,20]. The higher the elastic modulus, the higher the ability of the complex to compensate for the relative displacements by the elastic fibrin strains within the slices. The maximum distance travelled by the samples until the complete rupture of the fibrin filaments (RP) observed in the present study is consistent with the values of the elastic modulus reported in the literature. The RP for mucosa turned out to be significantly higher than that of bone and hard dental tissues, suggesting that the elastic component of the deformation might be greater in the presence of soft tissues [20].

The capability of mucosa to form a stable interface with the clot and to sustain tensile stresses reported in this in vitro study should prompt the development of flap and suture techniques that may exploit soft tissues in regenerative periodontal techniques. In periodontal plastic surgery, specific flap designs and suture techniques have already been proposed to displace the gingiva coronally and cover root exposition caused by gingival recession, thus restoring the normal appearance of the gingival margin, while, when the purpose is to regenerate the whole periodontal apparatus coronally to the bone crest, a scaffold is generally used to keep the soft tissue in a coronal position. A coronally advanced envelope flap associated with amelogenin and stabilized by single interrupted sutures has been proposed in the treatment of self-containing infrabony defects to improve the stability of the supracrestal soft tissue, preventing its collapse into the regenerative chamber, thus minimizing gingival recession and increasing the potential for clinical periodontal regeneration [21]. For the regeneration of non-self-containing intrabony defects, the “soft tissue wall technique”, in which the coronal advancement of a trapezoidal flap associated with amelogenin, followed by sling sutures for its coronal stabilization and an internal mattress suture, was experimented to achieve primary intention healing and closure of the papilla [9]. The significant improvement in clinical outcomes such as pocket depth reduction, clinical attachment level gain and recession reduction at one year of follow-up seems to support this surgical approach. One of the most challenging outcomes in dental practice is the regeneration of suprabony periodontal defects due to the absence of bony walls surrounding the regenerative chamber [22]. The results of the present study may lay the foundations for the implementation of incision and suture techniques relying on soft tissues alone for the anchorage and stabilization of the blood clot, providing stability to the mucosal wall and contributing to the creation of favorable conditions for regeneration to occur, possibly also resulting in non-self-containing defects where the bony wall stabilization role is limited or missing [9,23].

TS1 and TS2 may represent a measure of the adhesion between two slices of the same material, disregarding their elastic properties. The peak of TS detected during the DE (rTS2) represents the local maximum stress generated by the BCC in response to the mutual separation of the surfaces bound by the clot. The mean value of rTS2 was significantly higher for hard dental tissues than for bone and mucosa, while the rTS2 of bone tissue was significantly higher than that of mucosa. The corresponding value of stress detected soon after the coagulation phase (rTS1) showed a similar trend concerning hard dental tissues, thus suggesting a very strong adhesion within the blood clot–hard dental tissue complex. The detection of a TS increase 45 min after the start of coagulation (TS1) in all samples may reflect the physiological activity of the blood clot. Basically, after tissue’s injury and blood vessels’ damage, the activation of blood clotting mechanisms leads to hemostasis: the vessels spam and platelets form a plug. Following fibrin polymerization and fibrinogen-mediated platelet aggregation, which stabilize the blood clot, the clot contracts. The platelets’ intracellular interaction of actin and non-muscle myosin generates contractile forces that propagate through the fibrin network thanks to strong platelet–fibrin interactions, thus increasing the clot stability. Furthermore, the newly deposed fibrine network acts as a provisional matrix for cell migration into the wound site, contributing to tissue neoformation and its remodeling [24,25].

The morphology of the curves represented in Figure 4 indicates features of the bonding between the clot and the different substrates. The joint and interfaces can be characterized by their ability to absorb energy. The mechanical response is defined as brittle if it absorbs relatively small energy before fracture and failure occurs just after the peak. It can be defined as quasi-brittle if failure is characterized by the development of micro-cracks, which grow and coalesce to form macro-cracks, and a post-peak branch can be observed, endowed by the dissipation of energy. The strain at failure remains, however, limited. In the current study, hard dental tissues showed an overall response typical of quasi-brittle materials. On the contrary, the response of mucosa exhibited a clear nonlinear pattern due to its marked elasticity. The elastic stiffness of the sample represents the tangent of the response at the origin. For the mucosa, the overall response turns out to be nonlinear from the beginning, and a sharp peak is replaced by a wide plateau, followed by a slowly softening curve apt to dissipate a large amount of energy (referred to also as work of separation, per unit surface). This morphology refers to a strongly elastic tissue that exhibits large strains. Finally, the bone exhibited intermediate behavior, where the morphology of the curve was strictly related to the tissue micro-architecture, constituting quasi-brittle trabeculae. For bone tissue, the initial branch turns out to be approximately linear, the peak is not well marked and is characterized by a plateau and the final softening branch descends slowly. The oscillations exhibited by the plot can be related to the surface properties of the bone, in which the trabecular structure probably does not allow for uniform, homogeneous adhesion.

The limitations of the present study include the fact that our BCCs were made of two samples of the same material facing each other, while in the clinical practice, the blood clot interacts with different tissues. Further experiments should therefore involve complexes including combinations of different tissues. An additional limitation was the choice of the tested tissues: to simulate bone and gingiva, we used bovine cancellous bone and cheek’s mucosa due to ethical reasons and to the need of the availability of large amounts of tissue, while hard dental tissues were represented by dentin rather than cementum, as it was not possible to obtain a plane surface of this tissue. Anyway, it should be considered that cementum and dentin have a similar chemical composition, and often, during root-planning procedures performed throughout non-surgical or surgical therapy, the cementum is partially removed, and dentin may emerge [26,27]. Furthermore, compatibility between human and bovine tissues is not an issue, as biomaterials from bovine origin are largely used in humans [6]. Finally, the interpretations of our results from a clinical point of view, suffer from the limitations of in vitro models in general and of some specific issues, such as that stimuli on the blood clot–tissue complex other than a single orthogonal displacing force were excluded, while it is known that occlusal forces can deliver intermittent loads in axial and transverse dimensions on the tooth and be transmitted to the healing area [16].

## 5. Conclusions

To conclude, the results of the present in vitro study may lay the foundations for supporting the use of mucosa to act as a wall for regenerative procedures of suprabony periodontal defects, given its capability to form a stable interface with the clot. From a clinical point of view, this might imply the development and implementation of adequate flap designs and surgical techniques and the identification and definition of the characteristics of the defects that might benefit from this treatment option.

## Figures and Tables

**Table 1:** Mean and standard deviation (SD) of the normalized data of (a) the punctual and spontaneous TS generated spontaneously by the blood clot at the end of the BCT (rTS1), (b) the highest TS of the blood clot–tissue complex generated during the DE (rTS2), (c) the separation work generated during the DE (rAC), (d) the normal separation of the clot in terms of the distance (micrometers) travelled by the upper sample up to blood clot rupture (RP).

	Bone Mean (SD)	Hard Dental Tissues Mean (SD)	Mucosa Mean (SD)
rTS1 (gr/mm^2^)	0.2 (0.1)	0.8 (0.4)	0.004 (0.002)
rTS2 (gr/mm^2^)	0.2 (0.07)	2.0 (1.2)	0.1 (0.02)
rAC × 10^−3^ (gr/mm^2^)	65.1 (21.9)	132.2 (63.1)	54.7 (43.7)
RP (µm)	610.5 (265.5)	277.6 (108.1)	1351.5 (732.4)

## Data Availability

The raw data supporting the conclusions of this article will be made available by the authors on request.

## Supplementary Materials

The following supporting information can be downloaded at: https://www.mdpi.com/article/10.3390/medicina60101673/s1, Video S1: Debonding experiment performed with two hard dental tissue samples.
